# Empowering young athletes: the influence of autonomy-supportive coaching on resilience, optimism, and development

**DOI:** 10.3389/fpsyg.2024.1433171

**Published:** 2025-01-08

**Authors:** Na Zhang, Geng Du, Tao Tao

**Affiliations:** ^1^College of Physical Education and Sports, Beijing Normal University, Beijing, China; ^2^School of Sports Training, Wuhan Sports University, Wuhan, Hubei, China; ^3^Institute of Physical Education, Huzhou University, Huzhou, China

**Keywords:** autonomy-supportive leadership, resilience in sports, youth athlete growth, optimism, developmental pathways in sports

## Abstract

**Introduction:**

The present study investigates how autonomy-supportive coaching style influences youth athlete development through psychological resilience and dispositional optimism. Despite growing interest in factors that contribute to athlete development, gaps remain in understanding how coaching approaches interact with psychological traits to foster youth athletes’ growth. This study addresses these gaps by proposing a serial mediation model in which autonomy-supportive coaching indirectly enhances athlete development through resilience and optimism.

**Methods:**

Data were collected from 325 youth athletes and their coaches across training facilities and schools in China, and analyzed using structural equation modeling in SmartPLS.

**Results:**

Results indicate that autonomy-supportive coaching style significantly increases psychological resilience, which in turn boosts dispositional optimism, positively impacting athlete development. Both resilience and optimism serially mediate the link between coaching style and athlete growth.

**Discussion:**

These findings emphasize the importance of autonomy-supportive coaching in creating psychologically supportive environments that foster resilience, optimism, and developmental pathways in youth sports.

## Introduction

The development of athletes, particularly youth, has garnered considerable attention in the realm of sports science (Varghese et al., 2022). Preliminary research highlights the convoluted balance between physical training, mental preparation, and the overarching influence of coaching styles (Falcao et al., 2020; Varghese et al., 2022). According to recent studies, coaching style can profoundly impact not only the immediate performance outcomes of young athletes but also their long-term development and connection with the sport (Newman et al., 2023). This perspective is further supported by researchers who argue that coaching style emphasizing on autonomy support, constructive feedback, and a focus on skill development contributes significantly to the holistic development of athletes. This evolving understanding reinforces the crucial role that coaching style plays in the cultivation of a supportive and empowering coaching environment (Falcao et al., 2020), which is a significant precursor of athlete development among youth.

Although it has been theorized that coaching style can be associated with athlete development, limited research has empirically investigated these relationships, particularly linking autonomy-supportive coaching style with athlete development. The authors address this gap by assessing autonomy-supportive coaching style as an antecedent to athlete development. It is crucial to investigate coaching style in this context since a wealth of evidence indicates that supportive and empowering leaders influence a diverse range of individual attitudes and behaviors across various spectrums of life (Newman et al., 2023; Toprak, 2020; Wu et al., 2020). Moreover, coaching style can be practically addressed through appropriate interventions. Thus, identify coaching style as an antecedent may present opportunities to foster athlete development.

Prior literature has shown a dichotomy of youth sports programs pertaining to athlete development, highlighting their potential benefits as well as detrimental effects on youth athletes (Varghese et al., 2022). Research has shown that there are a variety of potential effects, including those in the areas of academics and professions (e.g., academic high achievers versus educational sacrifice), athletics and physical activities (e.g., improved physiological capacity versus injury), psychosocial issues (e.g., time spent away from family versus improved social skills, such as communication), and psychology (e.g., more confidence versus burnout) (Thompson et al., 2022). It is imperative to comprehend the holistic development impacts for youth athletes in intensified youth sports programs, given their popularity, the probability that most youth athletes will not succeed in their sport in the end, and the numerous and diverse positive and negative effects associated with these programs (Thompson et al., 2022). This duality suggests that individual psychological traits play a significant role in mediating the underlying mechanism.

Given this backdrop, we speculate that psychological resilience acts as a crucial mediator, empowering athletes to cope with the negative effects of high-demands sports environment. We anticipate that psychological resilience underpins the positive outcomes associated with autonomy-supportive coaching style. Athletes who are resilient are more likely to view constructive feedback as an opportunity for development, embrace challenges with a growth mindset, and develop a stronger sense of self-efficacy. Further, resilient individuals may culminate the impacts of coaching style not only to better equip their full athlete potential but also to promote their well-being and success beyond sports. Thus, by examining psychological resilience as a mediator between coaching style and athlete development, this study explores a finer-grain understanding of the underlying mechanism, linking leadership and individual psychological traits.

Subsequently, the study predicts that the influence of psychological resilience on athlete development is not a straightforward relationship, rather it is channeled through another mediating mechanism, i.e., dispositional optimism. Dispositional optimism refers to a consistent and enduring inclination to anticipate positive results in significant areas of life (Miranda and Cruz, 2020). In addition, dispositional optimism is linked to improved psychological adaptation to stressors, including both normative events like starting college and severe traumas [such as serious injuries or high-pressure competition] (Nes and Segerstrom, 2006). Thus, optimism complements the resilience trait of individuals to cope up with and bounce back from adversities. Accordingly, we anticipate that athletes who exhibit higher levels of psychological resilience are likely to possess or develop stronger dispositional optimism. This optimism, in turn, enhances their ability to adapt to the demanding nature of sports. Specifically, we predict that coaching style indirectly influences athlete development through the serial mediation roles of psychological resilience and dispositional optimism. Hence, this study presents an initial exploration into how psychological resilience and dispositional optimism serially mediate the relationship between coaching style and athlete development.

This study directly addresses the need for empirical clarity on how autonomy-supportive coaching style impacts long-term athlete development through specific psychological pathways. While prior research emphasizes the influence of coaching on immediate performance outcomes (Newman et al., 2023), limited empirical work has explored its extended effects on athlete development, particularly through the lens of psychological resilience and dispositional optimism. Recent studies suggest that coaches’ behaviors are significantly shaped by psychological antecedents, such as basic psychological needs, motivation, and subjective vitality, which together promote a more supportive and effective coaching approach (Inguglia et al., 2023). Given the significance of youth sports in fostering holistic athlete growth, understanding how autonomy support within coaching contributes to resilience is essential (Falcao et al., 2020; Wu et al., 2020).

Building on self-determination theory (Ryan and Deci, 2000) and resilience frameworks (Bajaj and Pande, 2016), this study explores the sequential mediating roles of psychological resilience and dispositional optimism. By examining these factors as a dynamic serial mediation pathway, the study extends the existing literature by illuminating the mechanisms through which autonomy-supportive coaching can nurture psychological traits that enhance athlete well-being and performance beyond the immediate sporting context. Additionally, recent findings emphasize the importance of coaches satisfying their athletes’ basic psychological needs as a foundation for promoting resilience and sustained engagement in sports (Inguglia et al., 2023). This research not only bridges a critical gap in understanding long-term development in youth sports but also provides actionable insights for designing coaching interventions that foster resilience, optimism, and subjective vitality, laying a foundation for sustained athlete growth.

### Research hypotheses

In the field of sports psychology, the concept of autonomy-supportive coaching has garnered significant attention for its impact on athlete performance outcomes (Bird et al., 2024). In juxtapose to conventional, directive coaching methods that prioritize control and uniformity, autonomy-supportive coaching style emphasizes the promotion of autonomy, understanding, and support for an athlete’s perspectives and needs (Hodge and Lonsdale, 2011). Theoretically, autonomy-supportive coaching is underpinned in the self-determination theory (SDT, Ryan and Deci, 2000), which posits that supporting an individual’s autonomy, competence, and relatedness needs fosters greater motivation, well-being, and performance. Empirical research has demonstrated the beneficial effects of autonomy-supportive coaching on athletes’ performance. For example, preliminary research by Mossman et al. (2022) has shown that athletes under the guidance of autonomy-supportive coaches exhibit higher levels of intrinsic motivation and psychological well-being. Furthermore, such coaching styles have been linked to improved team cohesion, athlete satisfaction, and persistence in sports activities (Lemelin et al., 2022).

Recent investigations have extended these findings to explore the specific pathways through which autonomy-supportive coaching style influences athlete outcomes. Our study speculates that autonomy-supportive coaching is positively linked to improved psychological resilience among youth athletes. Psychological resilience is “a personal trait that helps individuals cope with adversity and achieve good adjustment and development during trying circumstances. It is a trait that inoculates individuals against the impact of adversity and traumatic events” (Bajaj and Pande, 2016, p. 63). Autonomy-supportive coaching focuses on addressing the psychosomatic needs of the workforce (Mossman et al., 2022) and, as a result, promotes their well-being. Scholars have contended that the development of resilience in individuals can be facilitated by the impact of autonomy-support (Kegelaers and Wylleman, 2019). According to Lemelin et al. (2022), it has been found that it can increase the sentiments of happiness and control among followers. This, in turn, can enhance their positive psychology. Additionally, it can also help followers reach their maximum potential (Mossman et al., 2022). Prior research has provided empirical data demonstrating the impact of autonomy-supportive leadership on individual outcomes in the workplace, including job attitudes, organizational citizenship behavior (OCB), and performance (Shih et al., 2022). However, there was a lack of focus on the impact of autonomy-supportive coaching style on youth athletes’ psychological resilience. Accordingly, this study suggests that coaching style can have a beneficial impact on athlete psychological resilience. Hence, we propose:

*Hypothesis 1*. There will be a positive association between coaching style and psychological resilience.

We further expect that youth athlete’s psychological resilience fosters their dispositional optimism, which refers to “individual differences in generalized outcome expectancies” (Williams, 1992, p. 475). We build our argument on the corollary that sports impose high levels of competitive pressures on athletes (Falcao et al., 2020). However, individuals who draw on their resilient capabilities, are more likely to recover from the negative experiences more quickly (Kegelaers and Wylleman, 2019). This ability, in turn, nurtures a sustained positive outlook on life, which reinforces dispositional optimism. Similarly, according to Nguyen et al. (2016), psychological resilience engenders a proactive approach involving problem-solving technique to dealing with life’s challenges. This orientation enables individuals to deal with adversity as well as fosters a sense of agency and control over their circumstances (Ryan and Deci, 2000), ultimately, nurturing dispositional optimism. Moreover, the adaptive coping strategic orientation leveraged by psychological resilience leads to enhanced positive reframing and active coping, sustaining dispositional optimism. Hence, we propose:

*Hypothesis 2*. There will be a positive association between psychological resilience and dispositional optimism.

Subsequently, we predict that psychological resilience mediates the relationship between coaching style and dispositional optimism. As discussed above, autonomy-supportive coaching style aims to empower athletes and support their autonomy in order to ensure sustainable motivation (Mossman et al., 2022). This empowerment is one of the critical tools that provide athletes feelings of self-determination and autonomy so as to perform effectively. Therefore, they experience a sense of agency and control throughout the course of their practice (Ryan and Deci, 2000), which manifests as enhanced resilience, enabling them to more effectively confront stressors and setbacks. Subsequently, the perspective of agency and control fosters a positive outlook regarding their ability to influence their circumstances, thereby enhancing their optimism. Hence, we propose:

*Hypothesis 3*. Psychological resilience will mediate the relationship between coaching style and disposition optimism.

The notion that an athlete’s performance and optimism are related has a lot of conceptual merit based on face validity. For instance, it is easy to envision (or have firsthand experience with) the detrimental effects of a pessimistic athlete or the beneficial effects of an optimistic athlete on their performance. Relevant research supports this face validity, demonstrating that optimists are more likely to create a strategy for challenging circumstances (Maheshwari and Jutta, 2020), are less likely to give up (Nes and Segerstrom, 2006), and have a more upbeat perspective in stressful situations (Santana et al., 2023). In addition to face validity and pertinent and related research, there is additional direct empirical support that has connected optimism to productivity at work. For instance, in the healthcare profession, Bazargan-Hejazi et al. (2023) have found significant impacts of caregivers’ optimism on their stress, depression, and psychological well-being. In addition, in the occupational psychological context, researchers have associated optimism with improved job satisfaction and organizational commitment (Bangun et al., 2023). These arguments render support to our assumption that dispositional optimism leads to improved athlete development among youth. Hence, we propose that:

*Hypothesis 4*. There will be a positive association between dispositional optimism and athlete development.

Accordingly, we expect that dispositional optimism mediates the relationship between psychological resilience and athlete development. Prior research suggests that psychological resilience is a crucial precursor of individuals across a range of life spectrums (Lee et al., 2023), including youth sports. This orientation engenders individuals to recover quickly from difficulties, e.g., tough competition, injuries, or loss, etc., ultimately promoting their mental toughness. However, dispositional optimism reinforces the influence of psychological resilience on athlete development as it offers a stable expectation that *‘good things will happen’* (Ben Fatma et al., 2024), which catalyzes these potential benefits of resilience into tangible outcomes in athlete development. Hence, this supports our theoretical deduction that dispositional optimism mediates the relationship between psychological resilience and athlete development. Hence, we propose:

*Hypothesis 5*. Dispositional optimism will mediate the relationship between psychological resilience and athlete development.

**Figure 1:**
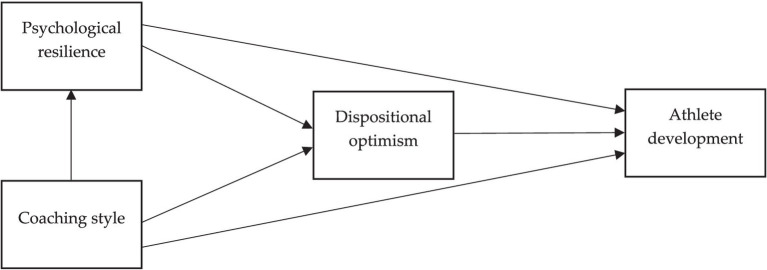
Conceptual model.

Hence, the aforementioned assumptions lead us to propose a serial mediation model encompassing psychological resilience and dispositional optimism as the serially mediated pathways between coaching style and athlete development, as illustrated in Figure 1. Hence, we propose:

*Hypothesis 6*. Psychological resilience and dispositional optimism serially mediate the relationship between coaching style and athlete development.

## Research methodology

### Participants

The study employed the stratified sampling technique to obtain a sample size of 325 for this investigation. Out of a total of 400 athletes and their coaches included in the survey, 364 respondents successfully submitted their responses at time 1 and 337 respondents delivered responses at time 2. Upon consolidation, 325 could match with the original responses, leaving a response rate of 81%.

To address potential common method bias (CMB) arising from self-reported measures, the study followed procedural remedies, such as using different sources for some variables, including self-rated scales for coaching style, psychological resilience, and dispositional optimism, while relying on coach-rated evaluations for athlete development. Further, collinearity was assessed using the variance inflation factor (VIF), with all VIF values remaining below the threshold of 3.3, indicating minimal risk of collinearity and bias.

In the demographic analysis, the study included a wide range of athletes. The age distribution of participants showed that 47% were between 12 and 15 years old. Those aged 16–18 years constituted 53% of the sample. The gender distribution constituted approximately 52% male and 48% female athletes. As for the types of sports, the sample was broadly divided with 32% participating in team sports, 24% in individual competitive sports, 19% in endurance sports, and the remaining 25% spread across other sports disciplines. Competitive level profiles indicated that majority of the participants were amateur youth athletes (70%) and 30% were in higher-level competitions with aspirations toward collegiate, national, or professional advancement.

### Instruments

The scale items are developed from the adapted versions of established measurement scales by employing a Likert 5-point measurement scale with values representing strongly disagree for 1 and strongly agree for 5.

### Coaching style

The coaching style construct was measured using the interpersonal behavior scale–the Echelle de Comportements Interpersonnels (ECI) developed by Otis and Pelletier (2000). The scale consisted of four items. An example of the autonomy-supportive coaching style scale items includes “My coach encourages me to find answers to problems I encounter during training.”

### Psychological resilience

The psychological resilience construct was measured using the adolescent resilience questionnaire (ALQ) developed by Gartland et al. (2011). The scale consisted of five items. An example of the ALQ scale items includes “I feel confident that I can handle whatever comes my way.”

### Dispositional optimism

The dispositional optimism construct was measured using the Life Orientation Test (LOT) developed by Scheier and Carver (1985). The scale consisted of eight items. An example of the LOT–dispositional optimism scale items includes “In uncertain times, I think the best.”

### Athlete development

The athlete development construct was measured using the scale items developed by Ford et al. (2011) and Kimbrough et al. (2007). The scale consisted of five items. An example of the athlete development scale items includes “This athlete effectively manages stress and focuses better during competitions.”

### Procedures

The study employs a cross-sectional research design using stratified sampling technique in order to assess the relationship between coaching style and athlete development through a serial mediation pathway encompassing psychological resilience and dispositional optimism. The stratification criteria included age, gender, sport type, and competitive level. By utilizing a wider sampling profile for our study, the study aims to ensure a representative of the broader athletic population.

Since all analyses are based on cross-sectional data, there are several implications for the findings. First, cross-sectional data capture relationships at a single point in time, restricting causal inferences. While associations between coaching style, resilience, optimism, and athlete development are observed, causation cannot be established. Future longitudinal research would be needed to validate these directional relationships over time. Second, although separating data collection waves and using coach ratings for athlete development aimed to reduce common method bias, cross-sectional designs remain prone to bias, especially in self-reported data. Participants might respond consistently across constructs, which can inflate observed relationships. Third, psychological resilience and dispositional optimism are dynamic traits that can evolve due to life experiences and coaching changes. Cross-sectional data provide only a snapshot, which may limit interpretations of resilience and optimism as stable mediators in the model. Longitudinal designs would better capture how these traits respond to coaching over time. Lastly, the findings may not reflect the ongoing and potentially changing impact of coaching style on athlete development across different stages of athletic growth. A longitudinal approach could clarify how coaching styles influence resilience, optimism, and development trajectories as athletes progress in their training and competitive careers. These limitations highlight the need for future research to adopt longitudinal designs to validate and extend the findings, allowing for a more comprehensive understanding of the dynamic effects of coaching style on athlete development.

Data were collected using structured questionnaires by employing a survey strategy. Respondents included young athletes and coaches at their respective training facilities or schools across China. The reasons for choosing athletes and coaches for participating in the survey was to avoid response biases in the survey, particularly attributed to self-rating or CMB. Coaching style, psychological resilience, and dispositional optimism scales were self-rated by the youth athletes at a given time. However, athlete development employed a coach-rating measurement scale. Further, both data collection waves were separated by a time interval of 2 weeks and specific instructions were provided in the cover letter along with the ethical and confidentiality assurance about how to generate keys to identify responses across waves.

### Data analysis

For data analysis, the study used a structural equation modeling (SEM) approach through SmartPLS software to evaluate both the measurement and structural models (Hair et al., 2019; Ringle et al., 2020). In the measurement model, reliability and validity were assessed by calculating Cronbach’s alpha, composite reliability, and average variance extracted (AVE) values for each construct, confirming internal consistency and convergent validity. Discriminant validity was examined using the Fornell–Larcker criterion and the heterotrait–monotrait (HTMT) ratio, both of which confirmed the distinctiveness of constructs in the model. The structural model was tested using the PLS algorithm with a bootstrapping technique (5,000 resamples) to determine the significance of path coefficients, including both direct and indirect effects. Through this process, the study evaluated the hypothesized relationships and the serial mediation effects of psychological resilience and dispositional optimism in linking autonomy-supportive coaching style to athlete development.

## Results

### Measurement model

The assessment of reflective measurement models involves three essential elements: average variance extracted (AVE) to determine convergent validity, reliability of individual indicators, and composite reliability to assess internal consistency. Discriminant validity is a key component in evaluating the reflective measurement paradigm. Researchers can evaluate discriminant validity by employing various methods such as the Fornell–Larcker criterion, cross-loadings, and specifically the heterotrait–monotrait (HTMT) ratio of correlations (Hair et al., 2019). At first, the internal consistency reliability was assessed by employing the Cronbach’s alpha and composite reliability measures. The reliability analysis demonstrates strong internal consistency across all constructs. Cronbach’s alpha values for coaching style (0.886), psychological resilience (0.888), dispositional optimism (0.879), and athlete development (0.901) all exceed the recommended threshold of 0.70, confirming reliable measures. Similarly, composite reliability values (rho_c) for each construct are above 0.90, indicating high internal consistency and reliability of the items. The average variance extracted (AVE) values for each construct—coaching style (0.745), psychological resilience (0.691), dispositional optimism (0.547), and athlete development (0.718)—are above the minimum threshold of 0.50, supporting the convergent validity of the constructs and affirming that the indicators adequately represent their respective latent variables. These results confirm that the measurement model is robust and suitable for further analysis (Table 1).

**Table 1:** Construct reliability and validity.

	Cronbach’s alpha	Composite reliability (rho_a)	Composite reliability (rho_c)	Average variance extracted (AVE)
Coaching style	0.886	0.888	0.921	0.745
Psychological resilience	0.888	0.893	0.918	0.691
Dispositional optimism	0.879	0.906	0.903	0.547
Athlete development	0.901	0.904	0.927	0.718

Discriminant validity, as defined by Hair et al. (2019), refers to the extent to which a construct is truly distinct from other constructs based on empirical criteria. In order to demonstrate discriminant validity, a construct must possess unique characteristics and have the ability to capture phenomena that other constructs in the model cannot capture. To assess the discriminant validity, we conducted cross-ladings analysis, which yielded values exceeding the minimum threshold and confirming distinctiveness. Additionally, we employed the Fornell–Larcker and heterotrait–monotrait (HTMT) ratio methods. Fornell–Larcker examines the relationships between the latent variables and the square root of the AVE values. For greater accuracy, the square root of the average variance extracted (AVE) of each construct must be higher than its highest correlation with any other construct (Hair et al., 2019). The discriminant validity of the results shown in Table 2 is confirmed by the Fornell–Larcker criterion.

**Table 2:** Fornell–Larcker.

	Athlete development	Coaching style	Dispositional optimism	Psychological resilience
Athlete development	0.847			
Coaching style	0.648	0.863		
Dispositional optimism	0.696	0.689	0.740	
Psychological resilience	0.626	0.522	0.717	0.879

Furthermore, Hair et al. (2019) propose the use of the heterotrait–monotrait ratio (HTMT) as a means of assessing the discriminant validity. HTMT is the term used to describe the ratio between within-trait correlations and between-trait correlations. The HTMT ratio has a maximum threshold value of 0.85. Our investigation, as shown in Table 3, produced results below this threshold, confirming the discriminant validity.

**Table 3:** Heterotrait–monotrait (HTMT) ratio.

	Athlete development	Coaching style	Dispositional optimism	Psychological resilience
Athlete development				
Coaching style	0.723			
Dispositional optimism	0.750	0.785		
Psychological resilience	0.679	0.571	0.748	

### Structural model

The objective of our study was to establish a connection between coaching style and athlete development through the intermediary roles of psychological resilience and dispositional optimism. Thus, following the successful validation of the measurement model, this study proceeds to evaluate the structural model. More precisely, the PLS method and bootstrapping approach were employed to test the structure routes and significant metrics (Hair et al., 2019). To evaluate the suggested model, both the direct and indirect effects were analyzed. The research demonstrates a significant and positive association between coaching style and psychological resilience. This is supported by the path coefficient value (*β* = 0.522), which indicates a significant influence (*p* < 0.01; *t* > 1.96). As a result, hypothesis 1 is validated. In addition, there is a significant association between psychological resilience and dispositional optimism. The path coefficient value (*β* = 0.491) demonstrates a substantial impact (*p* < 0.01; *t* > 1.96), confirming hypothesis 2. Similarly, there is a significant link between dispositional optimism and athlete development. This is supported by the path coefficient value (*β* = 0.310), which shows a significant effect (*p* < 0.01; *t* > 1.96). As a result, hypothesis 4 is validated.

**Figure 2:**
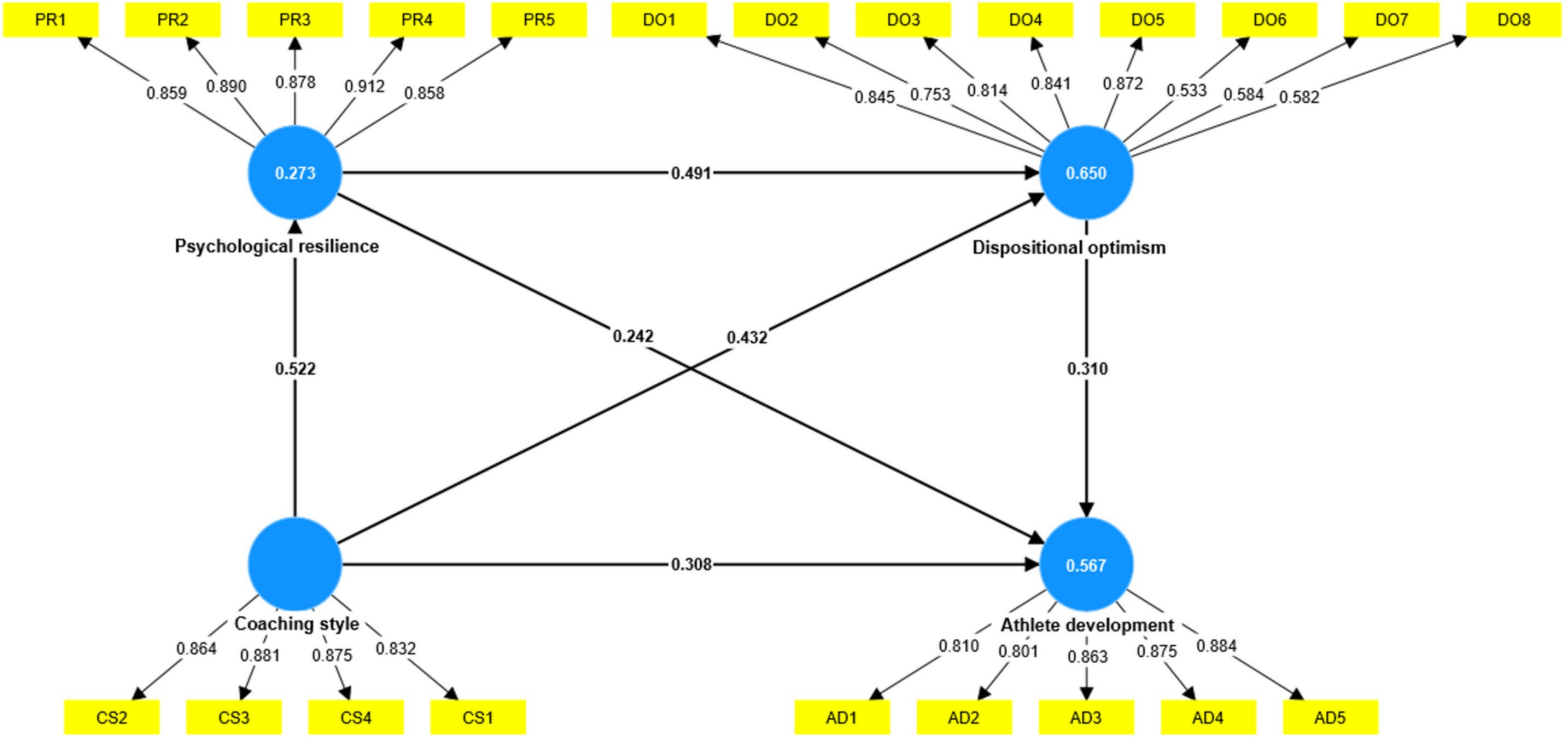
Structural equation model.

Furthermore, the empirical research verified the indirect correlations, using a resample of 5,000 bootstrapping procedures (Bias-corrected and accelerated). The study demonstrates that psychological resilience plays a major role in mediating the relationship between coaching style and dispositional optimism. The analysis revealed a path coefficient value of *β* = 0.257, indicating a significant influence (*p* < 0.01; *t* > 1.96). In addition, the study revealed that dispositional optimism plays a critical role in mediating the relationship between psychological resilience and athlete development. The path coefficient (*β* = 0.152) indicates a significant influence (*p* < 0.01; *t* > 1.96). Besides, the study has confirmed that psychological resilience and dispositional optimism plays a serial mediation role in the connection between coaching style and athlete development. The analysis showed a path coefficient value of *β* = 0.079, which had a significant effect (*p* < 0.01; *t* > 1.96). Furthermore, Figure 2 and Table 4 demonstrate that there is a significant direct correlation between coaching style and athlete development. This suggests that the proposed model involve complementary mediation.

**Table 4:** Path coefficients.

	*B*	*p*-value	*t*-value	Confidence intervals
Direct effects				
Coaching style → psychological resilience	0.522**	0.000	9.121	[0.400, 0.625]
Coaching style → dispositional optimism	0.432**	0.000	9.891	[0.340, 0.515]
Coaching style → athlete development	0.308**	0.000	4.844	[0.176, 0.428]
Psychological resilience → dispositional optimism	0.491**	0.000	11.923	[0.408, 0.572]
Psychological resilience → athlete development	0.242**	0.000	4.215	[0.135, 0.359]
Dispositional optimism → athlete development	0.310**	0.000	4.920	[0.185, 0.428]
Indirect effects				
Coaching style → psychological resilience → dispositional optimism	0.257**	0.000	7.880	[0.194, 0.320]
Psychological resilience → dispositional optimism → athlete development	0.152**	0.000	4.836	[0.092, 0.212]
Serial mediation effect				
Coaching style → psychological resilience → dispositional optimism → athlete development	0.079**	0.000	4.216	[0.045, 0.118]

Correlations are significant at 0.01 level (two-tailed).

## Discussion of findings and their implications

The current study examines the impact of coaching style on athlete development in the context of youth athletes in China and investigates how individual psychological traits, such as psychological resilience and dispositional optimism serially mediate the relationship between coaching style and athlete development. In order to examine the proposed framework, the authors employed a time-lagged, self-rated/coach-rated, research design by employing a sample of youth athletes in China. The study supports all six hypothesized relationships, demonstrating that autonomy-supportive coaching style significantly impacts athlete development through the serial mediation of psychological resilience and dispositional optimism. Specifically, coaching style was shown to enhance resilience, which, in turn, fosters dispositional optimism, ultimately contributing to youth athletes’ overall development.

While prior research has established links between coaching behaviors and athlete outcomes, our study’s serial mediation model involving resilience and optimism adds a unique perspective to the literature on autonomy-supportive coaching. Unlike studies focusing on immediate performance outcomes (Kegelaers and Wylleman, 2019; Lemelin et al., 2022; Santana et al., 2023), this research captures how supportive coaching indirectly fosters psychological traits crucial for long-term development. Additionally, implications extend to diverse athlete populations, including those with disabilities, where specific coaching styles may differently influence social and psychological development. For instance, Battaglia et al. (2019) examined the impact of a specialized aquatic program on adolescents with autism spectrum disorders, finding that structured coaching in a supportive environment significantly enhanced both social interactions and gross motor skills. These findings highlight the importance of adapting coaching styles to meet the unique needs of diverse populations, suggesting that autonomy-supportive approaches may be particularly effective in building both physical and psychological capacities. Our study broadens the potential applications of autonomy-supportive coaching by indicating that such styles not only support immediate performance but also cultivate resilience and optimism, traits essential for sustained growth and development across varied athlete populations.

Our results are in harmony with previous studies.

Specifically, our study found a significant positive impact of coaching style on athlete’s psychological resilience, accounting for 52.2% variation. Our study is in line with the extant body of knowledge on the leadership and psychological spectrums, as well as it extends the prior knowledge (i) by exploring the influence of leadership, i.e., coaching style in the realm of sports, on athlete’s psychological traits, and (ii) by embarking upon the autonomy-supportive coaching style as a prerequisite element in the process of athlete’s psychological and physical development. Previous studies investigating the relationship between leadership and resilience have demonstrated both contextual and conceptual disparities. For instance, Gaddy et al. (2017) investigated the influence of authentic leadership on subordinate resilience and found positive correlation. Similarly, Najam and Mustamil (2022) found that servant leadership has positive influence on subordinates’ psychological ownership and resilience. Moreover, Smith (2015) found positive associates between coaching and leadership resilience. Nevertheless, the exploration of coaching styles, especially the autonomy-supportive style, represents a novel and unique approach in assessing the psychological resilience of youth athletes.

The findings further indicate that psychological resilience plays a role in mediating the link between coaching style and dispositional optimism. There is a scarcity of research that examines the intermediary role of psychological resilience in the realm of youth athletes between coaching style and dispositional optional. The results of our study, thus, extend this omission in the academic discourse and align with previous literature. For example, Batool et al. (2022) discovered that psychological resilience mediates the relationship between servant leadership and organizational sustainability. In another study on a team level, Dimas et al. (2021) found that team resilience mediates the influence of transformational leadership on team effectiveness. The authors found that team resilience completely underpinned the impact of transformational leadership on team effectiveness.

Furthermore, the study discovered that the impact of psychological resilience on an athlete’s dispositional optimism transcends their psychological characteristics, influencing comprehensive development among the youth. This is supported by a significant mediation effect of dispositional optimism (15.2%) between psychological resilience and athlete development. The study not only complements existing research but also presents a contradiction to a body of literature that supports the notion that optimism fosters resilience. For instance, the relationship between optimism and resilience has been verified in several studies including Maheshwari and Jutta (2020) and Pathak and Lata (2018), among others. Conversely, aligning with the findings of our study, Souri and Hejazi (2014) conducted research that identified the positive impact of resilience on psychological well-being through the mediating role of optimism. However, only a few studies have delved into the relationship in a contrasting manner, thereby making our study a significant contribution to the relatively sparse body of literature in this context.

Specifically, our research expands to the extant body of knowledge in the wider spectrum of leadership styles, individual psychological predispositions, and development. Since the emergence of psychological resilience frameworks and their application in sports psychology, there has been an increasing academic and practical interest in understanding the factors that contribute to and result from such resilience in athletes (Simon et al., 2024). However, there has been scant research investigating the impact of coaching styles, particularly autonomy-supportive coaching, on the psychological resilience and subsequent development of youth athletes. By exploring the influence of coaching styles on athlete development, our study broadens the scope of existing research and contributes to the wider discourse on sports psychology and athlete development. Moreover, our research reveals that the relationship between coaching styles and athlete development in youth athletes is not straightforward; instead, it is facilitated by several intervening variables that mediate this relationship. Identifying positive psychological traits such as resilience and optimism as mediating factors, our study enriches the existing literature on positive psychology, and, more specifically, youth sports psychology (Bird et al., 2024). This enhancement to the academic discussion significantly progresses the theoretical, empirical, and practical understanding of our conceptual framework. Additionally, our findings suggest that the relationship between coaching styles and athlete development is further enhanced by incorporating practices that promote mental well-being and optimism among athletes. These findings are systematically analyzed and empirically validated through a mediation analysis, illustrating how factors like psychological resilience and dispositional optimism sequentially mediate the relationship between coaching styles and athlete development. Consequently, our research marks a pioneering examination of the nuanced pathways through which coaching styles impact athlete development through a serial mediation mechanism of psychological resilience and dispositional optimism.

### Practical implications

Practically, the findings of the current study suggest that an autonomy-supportive coaching style could serve as a foundational framework for the development of coaching training interventions aimed at fostering a thriving sports environment. When effectively implemented, such interventions have the potential to assist coaches in nurturing sports environments that are conducive to satisfying athletes’ basic psychological needs, fostering more volitional and autonomous behavior among athletes. This insight is anchored on the SDT (Ryan and Deci, 2000), which purports that satisfying basic psychological needs for autonomy, competence, and relatedness is imperative for promoting well-being and superior performance. Prior research in the occupational context suggests that autonomy-supportive leadership has substantial implications for employees’ positive outcomes. For instance, Hardré and Reeve (2009) conducted a study where they discovered that a training program focused on autonomy support led to an increase in autonomy supportiveness in management after 5 weeks. Additionally, this training program also resulted in higher levels of autonomous motivation and job engagement among the employees. Subsequently, our study found that coaching style significantly predicts psychological resilience and dispositional optimism, which in turn, translates into increased athlete development. Based on these findings, we suggest that training programs should be formulated and implemented that aim to equip coaches with the skills and strategies necessary to foster environments that enhance psychological resilience and dispositional optimism among athletes. Further, sports organizations and teams might consider incorporating principles of autonomy-supportive into their coaching philosophies and practices as a means to enhance athlete development, well-being, and performance. Ultimately, our model offers practical insights that could lead to a paradigm shift in coaching, with a greater emphasis on the integration of leadership and psychological aspects of athlete development.

### Limitations and recommendations for future research

Despite the significant strengths of this study, it is imperative to acknowledge its limitations. Primarily, the examination of data from a specific geographic region may limit the generalizability of the findings across diverse and polarized contexts. Secondly, the use of self-report instruments to examine coaching style, psychological resilience, and dispositional optimism raises the possibility that the study may be affected by common method variance and self-report measures (with an exception about athlete development that was coach-rated). Thirdly, our findings indicate clear evidence of serial mediating roles of psychological resilience and dispositional optimism between coaching style and athlete development. However, the proposed model did not assess the boundary conditions of the coaching style and athlete development linkage, for which we invite future studies to test moderators that could influence or impede these relationships. Lastly, we suggest future studies to control for all potential confounding variables that may limit stud’s ability to attribute findings directly to the variables of interest.

## Conclusion

Conclusively, the study examines the influence of coaching style on youth athlete development in Chinese contexts. Additionally, the study investigates the serial mediation roles of psychological resilience and dispositional optimism in these relationships. Our time time-lagged study has demonstrated that psychological resilience and dispositional optimism serially mediate the relationship autonomy-supportive coaching style and athlete development among youth. Our proposed model supported by empirical results not only extends the current academic debate on the leadership and psychological aspects of individuals in the context of youth sports but also offers noteworthy practically implications and future research avenues.

## Data Availability

The raw data supporting the conclusions of this article will be made available by the authors, without undue reservation.
