# Supplementary material for: Empowering young athletes: the influence of autonomy-supportive coaching on resilience, optimism, and development
Source: Front Psychol. 2025 Jan 8;15:1433171. doi: 10.3389/fpsyg.2024.1433171 (PMC11750835; doi:10.3389/fpsyg.2024.1433171)
Supplement: Supplementary file 2 [file Data_Sheet_2.docx]

**Appendix: Athlete Development Questionnaire**

**Coaching style: autonomy supportive**

1. My coach encourages me to find answers to problems I encounter during training."
2. "I feel that my coach listens to my ideas and considerations about training and goals."
3. "My coach provides me with choices and options in how I approach my training."
4. "My coach understands and considers my feelings and perspectives about my sport. "

**Psychological resilience**

1. "I feel confident that I can handle whatever comes my way."
2. "I think things through carefully before making decisions."
3. "I tend to think the worst is going to happen. (R)"
4. "I can express my opinions when I am in a group."
5. "I am patient with people who can’t do things as well as I can."

**Dispositional optimism**

1. "In uncertain times, I think the best."
2. "I'm always optimistic about my future."
3. "I hardly ever doubt that good things will happen to me."
4. "I expect to achieve most of the goals I set for myself."
5. "I rarely count on good things happening to me. (R)"
6. "It's difficult for me to expect things to go my way. (R)"
7. "Things never work out the way I want them to. (R)"
8. "I don't get my hopes up too high because I'm often disappointed. (R)"

**Athlete development**

1. "This athlete has significantly improved in my technical skills and physical condition."
2. "This athlete effectively manages stress and focuses better during competitions."
3. "This athlete’s understanding of game strategies and ability to make in-game decisions have enhanced."
4. "This athlete communicates more effectively with teammates and contributes to a positive team environment."
5. "This athlete feels more confident in his/her sports abilities and is closer to achieving his/her personal athletic goals."
